# Fibronectin and Hand2 influence tubulogenesis during pronephros development and mesonephros regeneration in zebrafish (*Danio rerio*)

**DOI:** 10.1371/journal.pone.0307390

**Published:** 2024-09-06

**Authors:** Lucia Carolina Uribe-Montes, Camilo Alfonso Sanabria-Camargo, Cristian Camilo Piñeros-Romero, Sebastián Otálora-Tarazona, Estefanía Ávila-Jiménez, Edwin Acosta-Virgüez, Zayra Viviana Garavito-Aguilar

**Affiliations:** 1 Laboratorio de Biología del Desarrollo–BIOLDES, Departamento de Ciencias Biológicas, Facultad de Ciencias, Universidad de los Andes, Bogotá, Colombia; 2 Departamento de Biología, Universidad Nacional de Colombia–Sede Bogotá, Bogotá, Colombia; NCMLS, Radboud University Nijmegen Medical Center, NETHERLANDS, KINGDOM OF THE

## Abstract

Worldwide incidence of kidney diseases has been rising. Thus, recent research has focused on zebrafish, whose fast development and innate regeneration capacity allow identifying factors influencing renal processes. Among these poorly studied factors are extracellular matrix (ECM) proteins like Fibronectin (Fn) essential in various tissues but not yet evaluated in a renal context. We utilized early *nat* and *han* zebrafish mutant embryos and carrier adults to investigate Fn’s role during kidney development and regeneration. The locus natter (*nat*) encodes Fn and the locus *han* encodes Hand2, which results in increased Fn deposition. Our results show that Fn impacts identity maintenance and morphogenesis during development and influences conditions for neonephrogenic cluster formation during regeneration. Histological analysis revealed disrupted pronephric structures and increased blood cell accumulation in Fn mutants. Despite normal expression of specification markers *(pax2*, *ATPα1a*.*1*), structural abnormalities were evident. Differences between wild-type and mutation-carriers suggest a haploinsufficiency scenario. These findings reveal a novel function for ECM in renal development and regeneration, with potential implications for understanding and treating kidney diseases.

## Introduction

The kidney is crucial for maintaining the body’s homeostatic balance through ionic and metabolite concentration regulation, waste removal, and blood pressure control. Studying mechanisms such as kidney development and regeneration can enhance our understanding of diseases affecting nephron function and help identify potential applications for tissue restoration. The zebrafish (*Danio rerio*) has emerged as a robust model in renal research. Molecular characterization of the zebrafish kidney anatomy has shown that the segment pattern and cellular composition in both embryonic stages and adults are comparable between zebrafish and other vertebrates, including humans [1, 2].

While zebrafish kidney development involves two stages—an initial transitory stage called the pronephros, which has an excretory function, and a final adult stage, the mesonephros—, mammalian kidney development includes an additional maturation stage, the metanephros, which forms the adult organ [3]. Unlike mammals, the zebrafish mesonephros exhibits neo-nephrogenesis, generation of new nephrons, throughout its life. This allows it to respond to physiological demands and restoring affected nephrons through tissue repair [4–6]. Despite structural and organizational differences among species, genetic pathways are highly conserved [7]. This makes the zebrafish an appropriate model for identifying novel mechanisms, genes, and other factors involved in development, regeneration, and renal progenitor regulation [8].

Pronephros development is accomplished by an initial renal field specification process followed by progenitor cell epithelialization and tubulogenesis. Subsequently, tubule segmentation occurs, ending with the formation of the glomerulus and inter-renal gland [9]. Key molecules associated with these stages include morphogens such as BMPs, Retinoic Acid, Nodal, and FGFs, transcription factors like *pax2*, *six2*, *osr1*, and components of the cellular microenvironment such as extracellular matrix (ECM) components.

The ECM provides an environment that modulates migration, signaling, and cell access to different regions, contributing to cell architecture, development, proliferation, polarity, and survival. This environment is dynamically regulated to respond to cell needs in a regenerative context [10, 11].

Fibronectin (Fn) is an extracellular matrix and basal membrane glycoprotein of vertebrates. It is the most abundant multi-adhesive matrix component and facilitates signal transduction across the plasma membrane [10, 12]. Fn plays an essential role in several developmental processes, including, gastrulation, precursor cell migration, adhesion, proliferation, differentiation, and tissue repair [11, 13]. It has been identified in cardiac regeneration [12, 14], and in the regeneration of skin, cartilage, and myelin [15], stimulating the recruitment of inflammatory and regenerative cells. The cellular microenvironment established by the matrix and its components like Fn provides physical and molecular tissue-specific signals [16]. The importance of this protein has been evidenced in organogenesis during zebrafish cardiac development, influencing the migration of cardiomyocytes towards the midline, with aberrations in the fusion process [11]. Additionally, Chou et al. determined that in zebrafish, Fn is essential for the correct positioning of the inter-renal gland. This gland is formed from bilateral populations of the intermediate mesoderm that migrate towards the midline on an Fn mesh secreted by the endothelial vasculature. The intimate association of this gland with the glomerulus and vascular tissue is also demonstrated [17]. However, the potential role of Fn in the specification of renal progenitors, epithelialization, tubulogenesis, and its influence in renal regeneration remains unknown.

In this research, we use a mutant loss-of-function approach to study Fn’s influence on the zebrafish renal scenario. It has been described that in zebrafish, the locus natter (*nat*) encodes for Fn and its *nat*^*tl43c*^ mutant allele has mainly been characterized in a cardiac context. The *nat*^*tl43c*^ mutation consists of a transversion in the *fn1* gene that inserts a translational stop signal at codon 81. The mutant phenotype is characterized by a flattened hindbrain and several intermediate cardiac phenotypes, ranging from bilobulated ventricles to cardia bifida (two heart formations separated in lateral positions) in more severe cases [11, 18].

Fibronectin protein levels can also be limited by a lateral plate mesoderm-expressed transcription factor, Hand2. There has been reported an inverse relationship between *hand2* and *Fn* function, where *hand2* (*han*^*S6*^) mutants present augmented Fn expression and a similar, more severe cardiac phenotype [18]. *Han*^*S6*^ is a null allele with a complete lack of *Hand2* gene product. This transcription factor limits kidney size by repressing intermediate mesoderm formation, establishing a barrier of lateral limits, and promoting the formation of venous progenitors [19, 20]. While Perens et al. observed lateral tubule expansion in *hand2* loss-of-function mutants, derived from an increased number of tubule cells, a subsequent role in pronephric tubulogenesis is yet to be determined [20].

We report the first evidence of the effects of *Fn* and *hand2* loss-of-function mutants on the progression of tubulogenesis during pronephric development and the mesonephric regeneration process. We show the absence of some segments of the pronephric structures in Fn mutants, where cells are unable to undergo effective tubulogenesis or lumen formation. We suggest that this outcome is not associated with the specification of renal progenitors or the segmentation process. *Hand2* mutant embryos also showed morphogenetic and structural alterations in enlarged pronephric tubules.

Both homozygous mutants are embryonic lethal given the dramatic phenotypes. Embryos do not survive beyond 5 days post-fertilization, making it impossible to perform regenerative studies using homozygous adults. However, adult heterozygous mutants, through anatomical analysis showed consistent tubule characteristics similar to those observed in homozygous mutant embryos, a phenotype that has not been reported before.

Our results provide evidence of Fn’s influence on renal tissue structure and organization, likely linked to several important and complementary mechanisms during development and regeneration, such as cell polarity and subsequent activation of additional extracellular matrix factors.

## Results

### Fibronectin participates in the correct pronephros organogenesis

**Fig 1:**
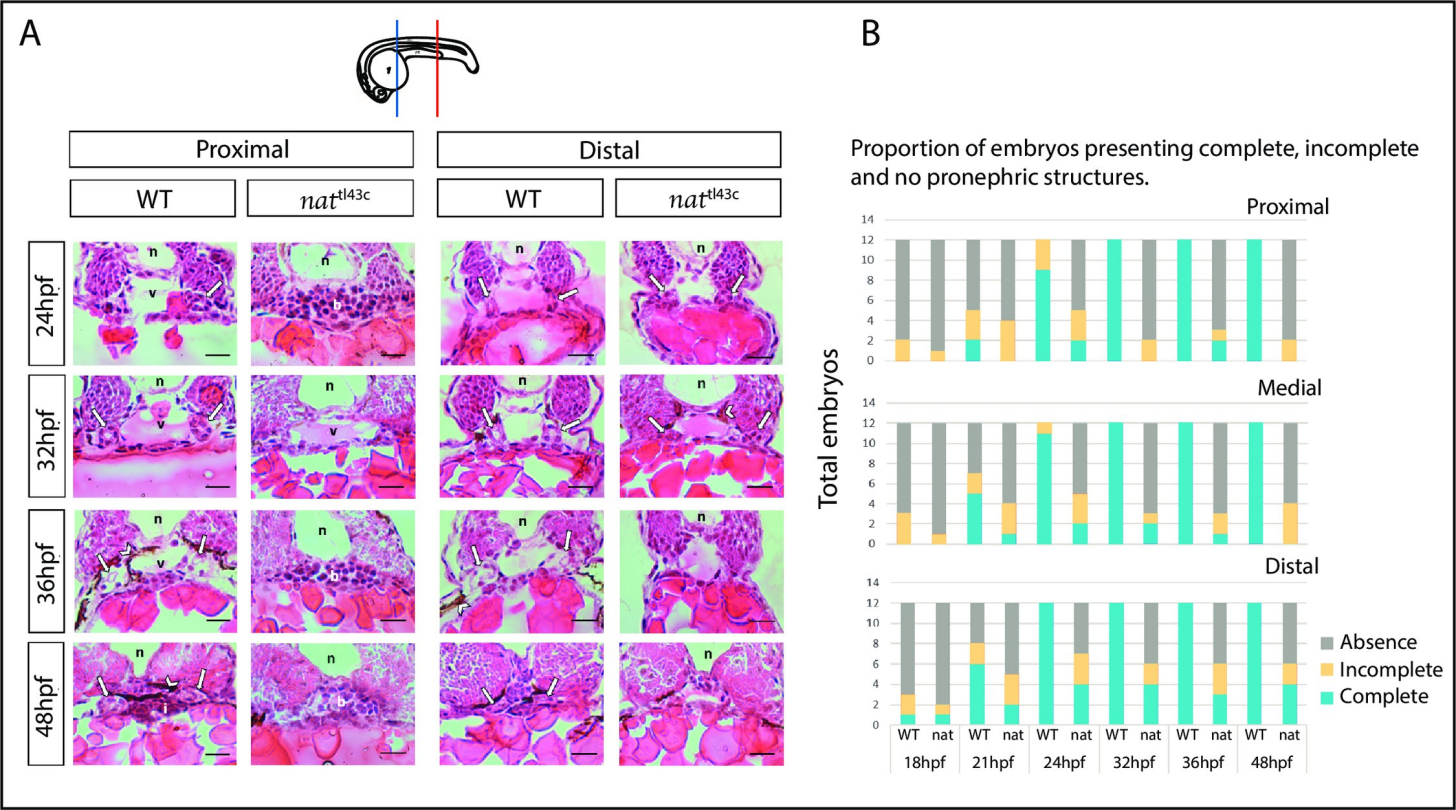
*nat^tl43c^* mutants have a pronephric phenotype. **A.** Transversal H&E histological slides for Fn mutants and their WT siblings at proximal and distal levels. From 18 to 21 hpf no pronephric structures are visible. At 24, 32, 36, and 48 hpf, WT embryos pronephric structures (white arrows), as well as normal vascular structures (v) are noticeable. By 48 hpf WT embryos have normal intestinal lumen (i). Mutant embryos do not present normal vasculature or pronephric tubules, and by 48 hpf, mutants show poorly defined intestine and vasculature. Moreover, WT embryos show a great amount of melanocytic neural crest cells (arrowheads) whereas mutants have an exacerbated amount of blood cells. In mutants, the pronephric structure appears more frequently in distal levels than proximal levels, but they are poorly defined. **n** (notochord); **v** (vasculature); **b** (blood cells); **i** (intestine). Dorsal up. Scale bar: 15 μm. **n = 12** from three independent experiments (4 embryos each). The diagram shows the level examined: blue for proximal and red for distal. **B.** The proportion of embryos showing complete (bilateral appearance of evident and easily recognizable pronephric tubules), incomplete (absence of one structure), or total absence (no discernible or absence) of pronephric structures in three distinctive anatomical locations (proximal, medial, distal) as examined in H&E histology**. n = 12**.

Basic hematoxylin-eosin (H&E) histology of embryos indicated the existence of a renal phenotype associated with the *nat*^*tl43c*^ mutant allele. The analysis was performed at 18, 24-, 32-, 36-, and 48-hours post-fertilization (hpf), along the anteroposterior axis at proximal, medial, and distal pronephric regions. By 18 and 21 hpf, there was no evident pronephric structure in mutant embryos and their WT siblings, as expected at these stages (S1 Fig). By 24 hpf, lumen formation was practically complete in WT embryos. However, in the *fn1* mutant embryos, the pronephric structure was absent in several cases for this and the subsequent stages analyzed (Fig 1A). In most 24 hpf mutant embryos, there was no morphological evidence of normal pronephric tubules, and vascular structures were poorly defined compared to WT. Additionally, cell disorganization was found in the somitic and notochord tissues of most mutants. There were no significant differences between proximal, medial, and distal regions in mutants (S1 Fig); however, the frequency of pronephric tubular appearance was slightly increased in the most posterior regions.

The phenotypic appearance was classified as a complete, incomplete, or total absence of pronephric structures. Bilateral and easily recognizable pronephric tubules were considered as complete, while the presence of only one recognizable structure in the embryo was considered incomplete (Fig 1). Visible pronephros structures in mutants were poorly defined, and the cell number composition was reduced at all levels relative to WT. Additionally, in mutants at the distal level, the cell number tends to increase relative to their proximal portions and over time from 18 hpf to 24 hpf (S1 Fig). Furthermore, a significant accumulation of blood cells was evident in mutant embryos compared to WT (Fig 1). All these results demonstrate that protein Fn1 is required for the proper pronephros organogenesis.

### Specification, determination, and segmentation markers express normally in *nat*^*tl43c*^ mutants, but organization and lumen formation of pronephric cells is affected

**Fig 2:**
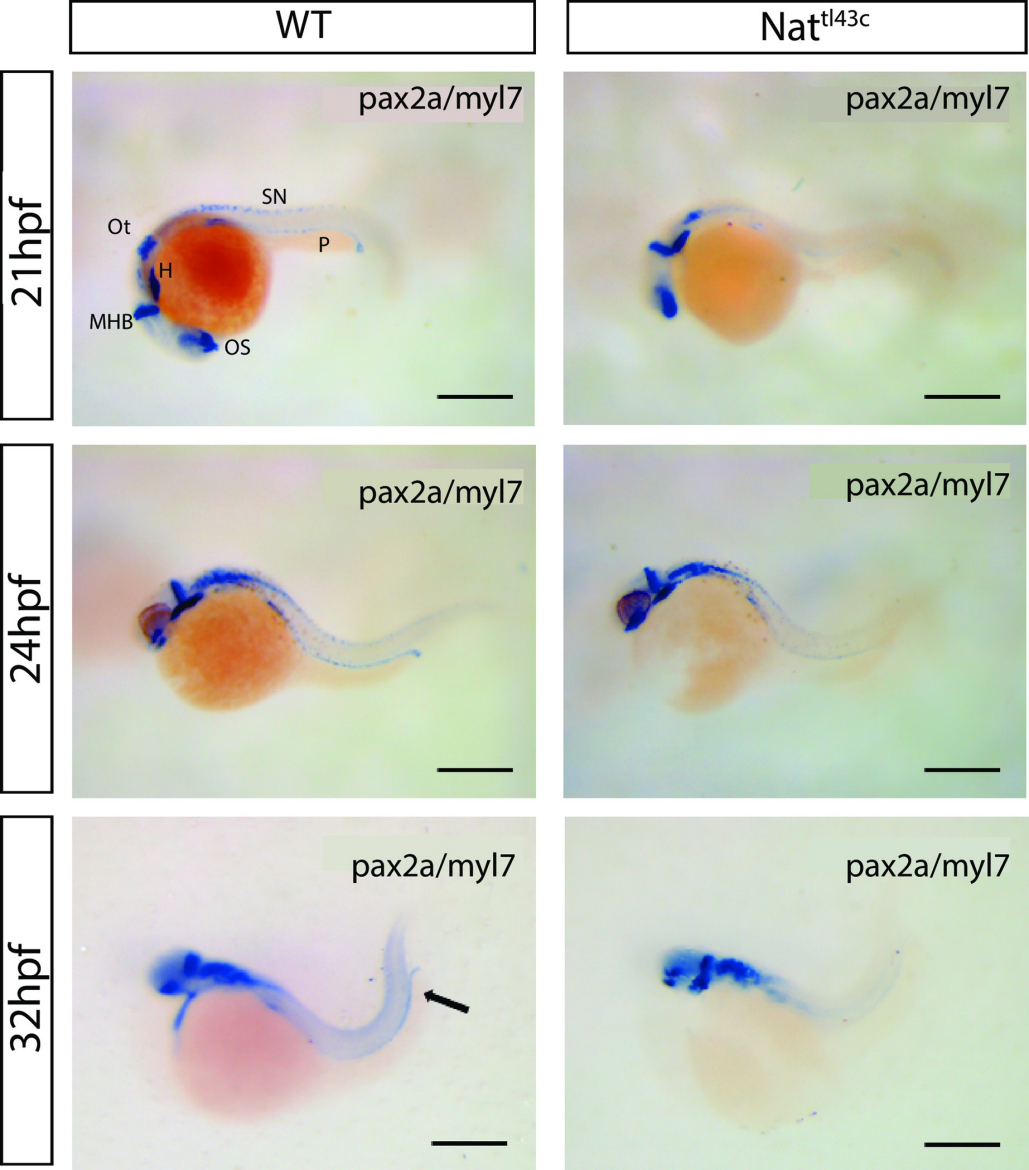
*pax2* gene expression in *nat^tl43c^* mutants and WT. Double in situ hybridization for *pax2* (renal field) and *Myl7* (heart field) genes. Even though there is a weaker signal, the expression of *pax2* marker is visible in WT as well as in mutants. **SN** (Spinal cord neurons), **P** (Pronephros), **Ot** (Otic vesicle), **H** (Heart), **MHB** (Midbrain to Hindbrain Boundary), **OS** (Optic stalk). Dorsal up, anterior to the left. Scale bar: 300μm.

To determine whether the observed abnormalities of tubular pronephric structures in mutants could be explained by a disruption in the specification or determination of renal progenitors, we evaluated the expression of two markers *pax-2* and *myl7*. *In situ* hybridization for the early specification marker *pax-2* gene shows a normal expression in the pronephric field of *nat*^*tl43c*^ mutants. Additionally, there were no appreciable differences in other expression domains, such as the otic vesicle, midbrain to hindbrain boundary, or optic stalks (Fig 2). The *myl7*, cardiac exclusive marker, allows us to confirm the characteristic cardiac phenotype in *nat*^*tl43c*^ mutants.

**Fig 3:**
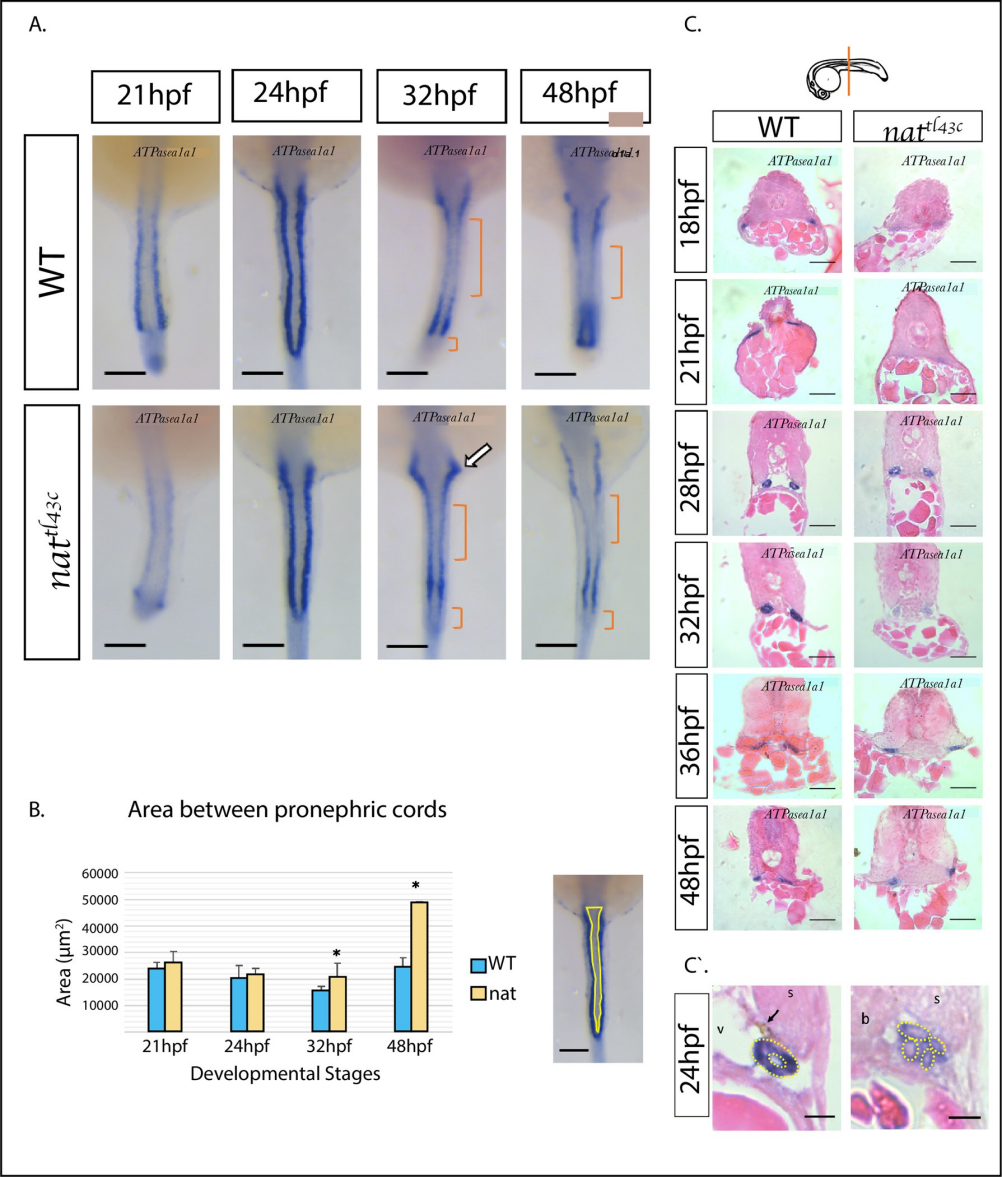
Renal cells determination occurs normally in *nat^tl43c^* mutants. **A**. The pattern of expression from 21 to 48 hpf of Myl7 / *ATPα1a*.*1* gene *in situ* hybridization. There is no major difference between the expression of WT and mutant embryos. At 32 and 48 hpf, the expression signal is localized at the anterior and medial region of the renal field (brackets), indicative of tissue segmentation. The arrow shows the presumptive Proximal Convoluted Tube (PCT). **B.** As indicated. **n = 8,** measurements were done on two independent hybridization experiments. Results presented as mean ± s.e.m. p<0.05. Dorsal view, anterior up. Scale bar: 100 μm **C.** H&E histology slides on *ATPα1a*.*1* hybridized embryos. Although in most cases the pronephros was absent in mutants, they have renal cells determination as can be evidenced by the *ATPα1a*.*1* expression signal. Dorsal up. Scale bars: 15 μm. n = 4. Diagram shows the level examined. **C`.** H&E histology slides on *ATPα1a*.*1* hybridized embryos at 24 hpf show that cells are determined to renal fate but show epithelial abnormalities. WT embryos present normal organized pronephric cell clusters with a lumen and normal anatomical characteristics like Dorsal Aorta (v) and few melanocytic neural crest cells (arrow). *nat*^*tl43c*^ mutants show disorganized pronephric cells that fail to form lumen and a lower expression signal intensity. There are no melanocytic neural crest cells and there is blood cell accumulation. s (somite), v (vasculature), b (blood cells) Left side, Dorsal up. Scale bars C`: 4 μm.

The renal determination marker *ATPα1a*. is a specific renal field marker whose protein product has important physiological and signaling functions in the nephron. Monitoring its expression over time provided important information about the state of the renal field state and its progression in development. Whole-mount *in situ* hybridization for *ATPα1a*.*1* gene shows continuous expression along the renal field until 24 hpf (Fig 3A) in both WT and mutants. By 32 hpf, *ATPα1a*.*1* continuous expression is interrupted for both WT and mutants. The signal strongly localizes in specific renal segment domains (anterior and distal regions) while it is considerably diminished in others (medial and ductal regions) (Fig 3A brackets). The specific localization of the signal is characteristic of the segmentation process. Thus, our observation suggests that renal cells determination and the normal process of segmentation are independent of Fn, and the morphogenetic process of tubulogenesis.

A more abrupt convolution in the presumptive *proximal convoluted tubule* (PCT) can be observed in mutant embryos at 32 hpf (white arrow in Fig 3) compared with the WT siblings. By 48 hpf, the restricted localization of *ATPα1a*.*1* signal is more evident. There is also increased space between the pronephric cords in the most anterior region of the 48 hpf mutant embryos, as well as a possible increase in the anteroposterior length as seen in the image (Fig 3A). To confirm this, we measured renal field lengths and areas along the anteroposterior axis and between the pronephric cords to determine the effect of loss gene expression of *fn1* on renal field size according to *ATPα1a*.*1* expression pattern (Fig 3B). The renal field length in both WT and mutant embryos shows a slight increase over time with no significant differences from 21 to 32 hpf (S2 Fig). Interestingly, by 48 hpf, the renal field length (Fig 3B) and the inner area measurements between pronephric cords show a significant increase in mutants. The anterior extension of pronephric cords and the separation between them account for the double increase in the area measured in 48 hpf *nat*^*tl43c*^ mutants compared with WT.

When we examine cross-section histological slides, we confirmed continuity of *ATPα1a*.*1* expression. However, we observed the absence of some tubular structures in *nat*^*tl43c*^ mutants and a generally disorganized cellular architecture (Fig 3C). A detailed view at 24 hpf embryos slides, shows that while pronephric cells in WT embryos exhibit an organized cluster forming lumen, mutant embryos are disorganized and fail to form a tubular structure (Fig 3C).

### *Hand2* loss-of-function mutations exhibit differential morphogenetic features in renal and vascular development

**Fig 4:**
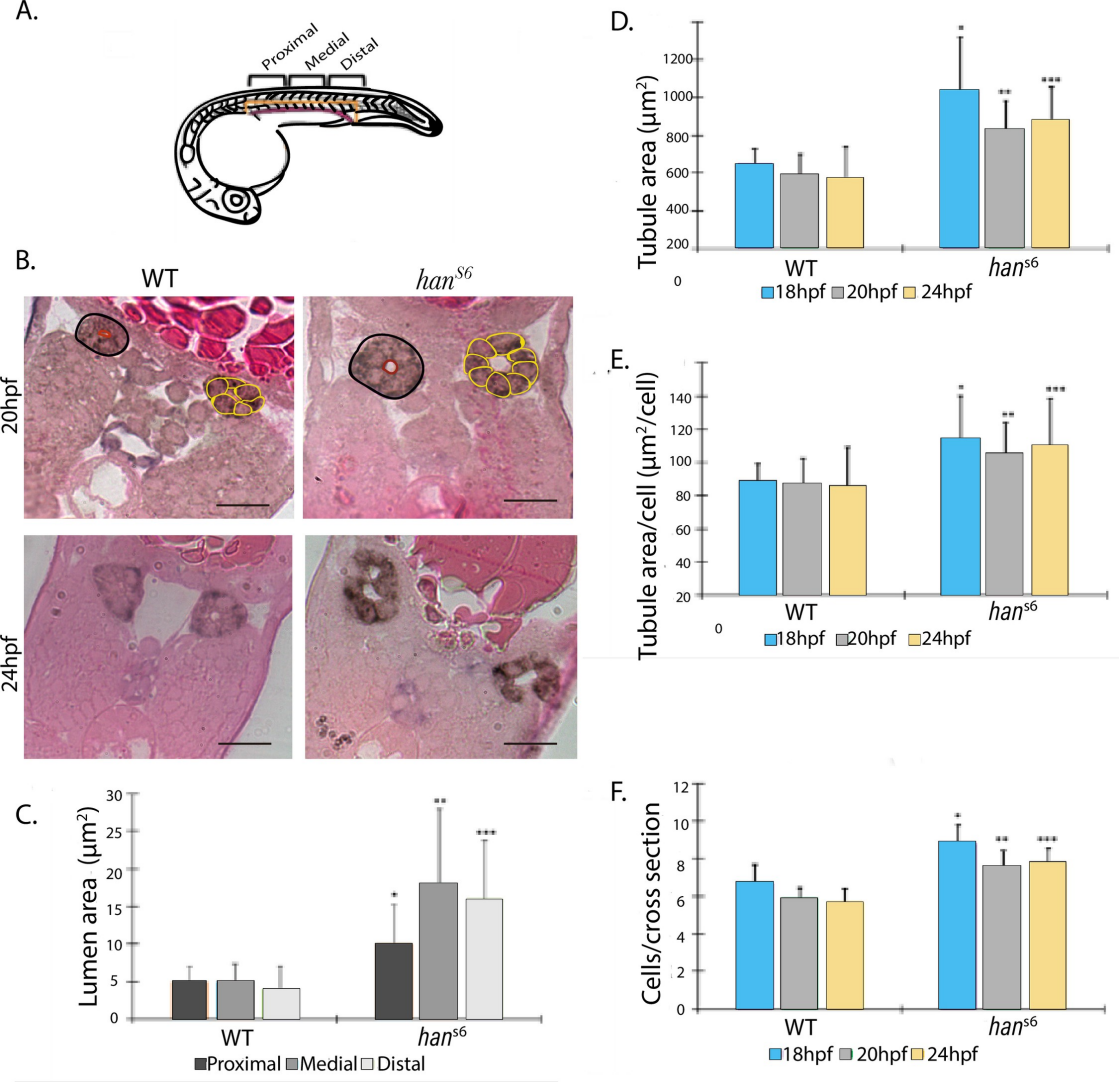
Cell and tissue characteristics of developing pronephric tubule in WT and *han^s6^* embryos. **A.** Schematic of a 24hpf zebrafish embryo. Orientation of tissue sectioning was perpendicular to the A-P axis. B. Representative images of observed patterns at 20 and 24 hpf. Black circles indicate demarcated area of the tubules, red circles show the lumen and yellow circles show cells that constitute the tubules. It is possible to observe a higher cell number of cells, bigger tubule, and lumen areas in *han* mutants. **C** Lumen area differences at 24 hpf were significant along the A-P axis (*n = 18*; *p-value≤ 0*.*001;* Dunn’s *post-hoc* test*)*. **D.** Tubule area differences between the two groups were significant at each evaluated stage (*n = 18*; *p-value≤ 0*.*001;* Dunn’s *post-hoc* test*)*. **E**. The calculated index tubule area per cell number showed significant differences between pairs of categories at all development stages (*n = 18*; *p-value≤ 0*.*001;* Dunn’s *post-hoc* test). **F** Cell -counts per cross-section per stage showed significant differences between WT and *han*^*s6*^ embryos (*n = 18*; *p-value≤ 0*.*001;* Dunn’s *post-hoc* test*)*.

We explored cellular and tissue changes in IM and LPM adjacent progenitors during pronephros tubulogenesis in the context of *hand2* loss-of-function by analyzing of semi-serial sectioning of WISH-processed embryos (Fig 4).

We examined semi-serial sections and found a correlation between the progression of pronephric tubule development and several morphometric parameters. Four different morphometric parameters showed statistical differences between WT and *han*^*s6*^ embryos, with higher values in mutant embryos for: Tubule lumen area (Fig 4B and 4C) and tubule cells number (p = < 0.001) at 24 hpf; section tubule area (Fig 4B and 4D) at 18, 20, and 24 hpf; the defined ratio between tubule section area and cell number per section (Fig 4E) and in renal cells number per tubule (Fig 4B and 4F).

**Fig 5:**
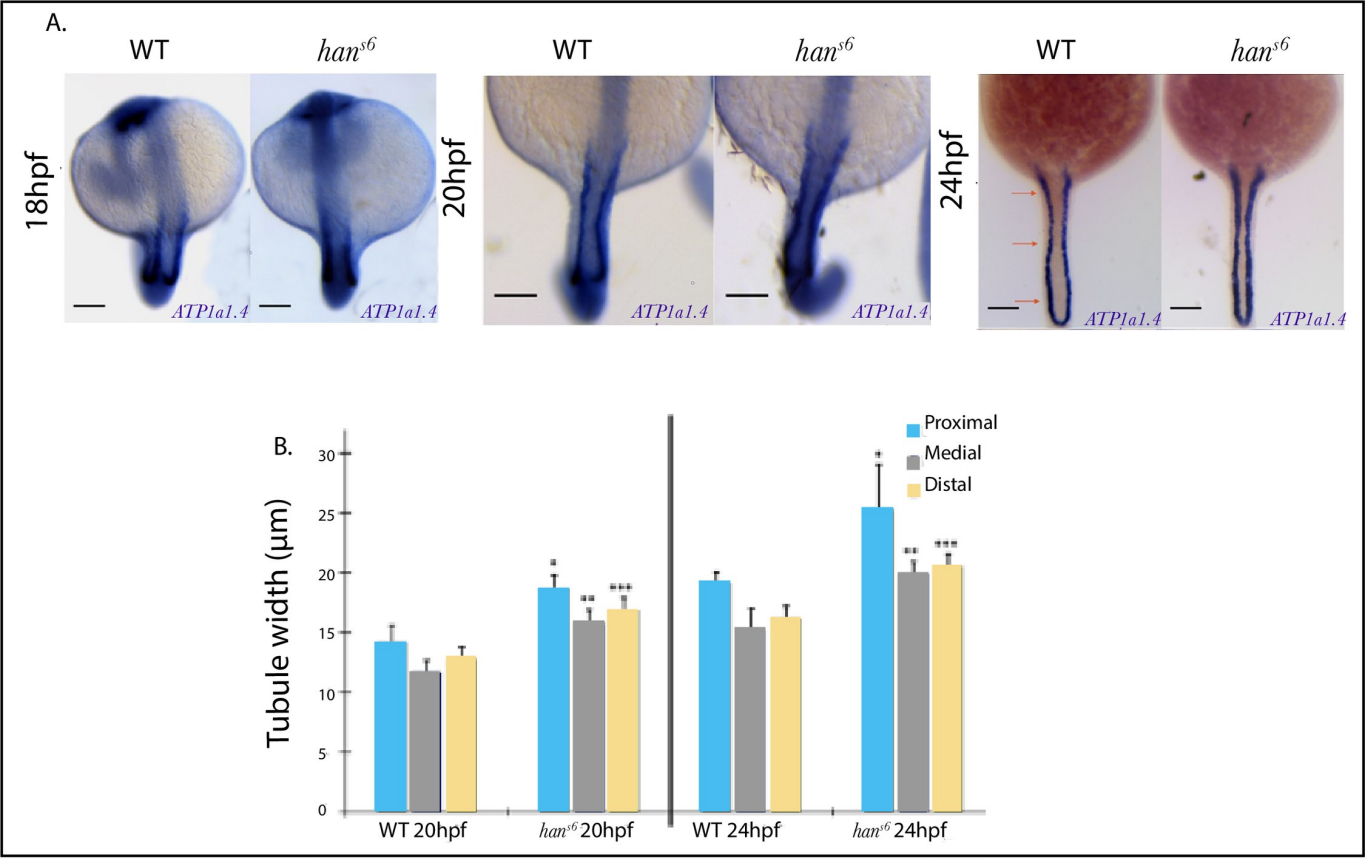
Comparison of *ATP1a1a*.*4* pronephric tubule expression patterns during late somitogenesis of WT and *han^s6^* embryos. **A**. *ATP*1a1a.4expression within the pronephric tubule at 24 and 20 hpf, illustrating the lateral expansion in *han*^*s6*^ mutants. At 18 hpf, this difference is barely noticeable. Scale bars: 100 μm. **B**. Pronephric tubule width measurements of WT and *han*^*s*6^ mutants taken at three defined levels along the A-P axis. Significant differences were found between pairs of levels compared in all plotted stages (*n = 7*; *p-value*≤ 0.005; Dunn’s *post-hoc* test).

To corroborate our observations at the whole organism level, we characterized pronephros development through WISH detection of *ATP1a1a*.*4* in WT and *han*^*s6*^ embryos at 18, 20, and 24 hpf (Fig 5). These results allowed us to evaluate the relationship between the presence of *hand2* and pronephros epithelization throughout late nephric tubulogenesis.

At 18hpf, no differences were found (p *=* 0,811). However, at 20 and 24 hpf, we observed a wider lateral expression in *han*^*S6*^ mutants (Fig 5A). This difference was significant at multiple levels along the anterior-posterior axis (Fig 5B).

The anteroposterior pronephric length (S3 and S4 Figs) and the distance between the otic vesicle lateral edges did not differ between WT and *han*^*s6*^ embryos, indicating the absence of non-specific global morphogenetic defects in development elicited by *hand2* abrogated expression. In summary, our results at the whole organism level validate the role of *hand2* as a specific limiting factor of pronephric tubule lateral dimensions.

Overall, our results constitute a novel quantification of *hand2´s* role in pronephric tubule and vascular morphogenesis, focusing on its activity on the posterior IM and LPM derivates.

### Pronephric tubule structure and morphogenetic alterations prompted by *hand2* loss-of-function

We further characterized the progression of tubulogenesis, distinguising three levels along the A-P axis, corresponding to proximal, medial, and distal.

**Fig 6:**
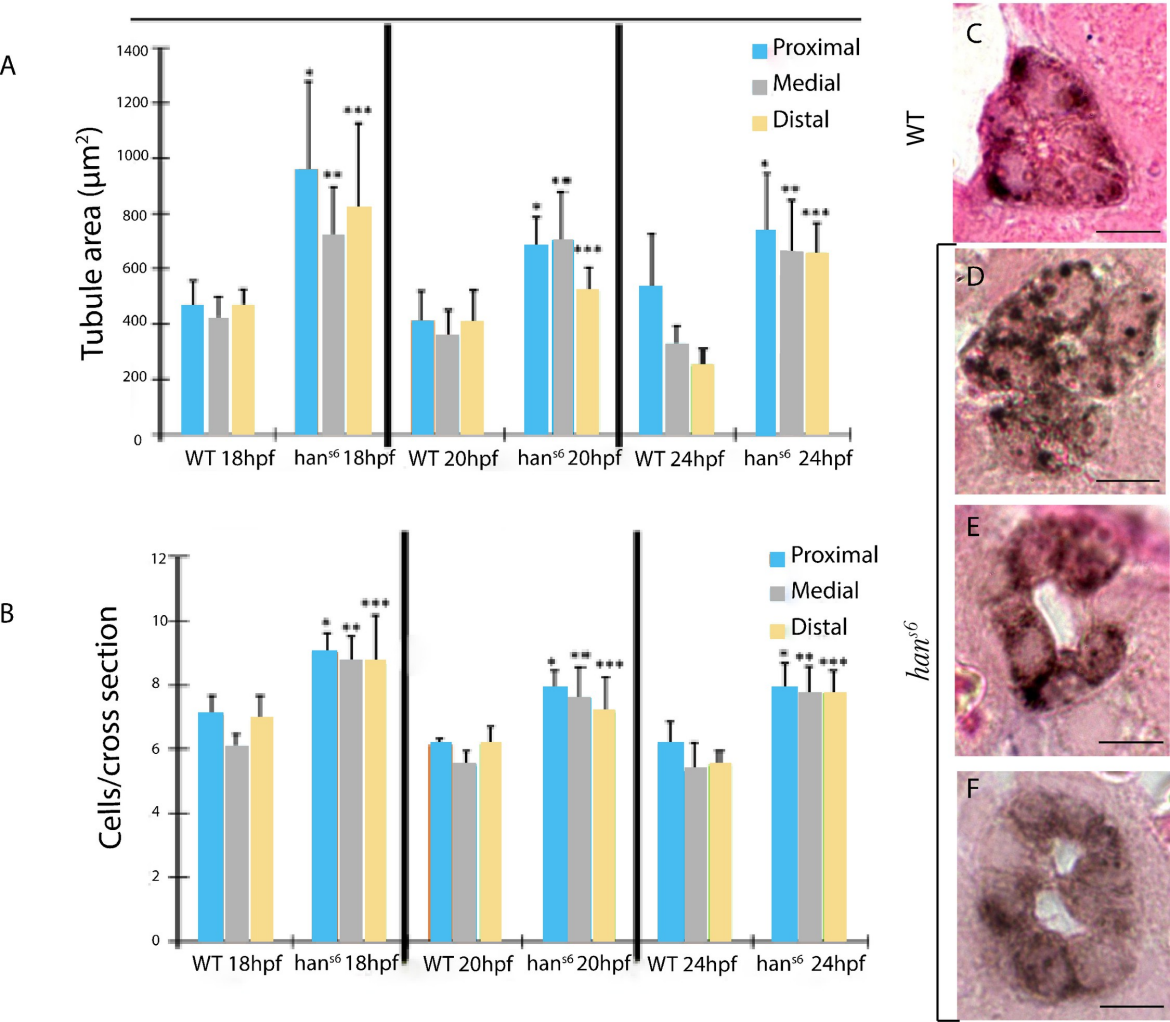
Comparison of morphometry parameters and tissue organization in WT and *han^s6^* sections (along the anteroposterior axis) during tubulogenesis. **A.** Pronephric tubule area measurements and **B**, cell number per cross-section from 18 to 24 hpf in WT and *han^s6^* embryos at three levels along AP axis. Significant differences were found between pairs of levels evaluated by phenotype (*n = 18*; *p-value≤ 0*.*005;* Dunn’s *post-hoc* test). Diverse defective settings of pronephric tubule organization were observed in comparison to the compacted cell assembly in WT embryos (**C**), such as erroneous location of cells outside the area of lumen formation (D), loss of tubule structural integrity (**E**), and two visible lumens (F) Scale bars: 20 μm. Brown staining corresponds to *ATP1a1a*.*4* expression pattern in pronephric tubule.

In WT embryos we observed a reduction in cell number throughout tubulogenesis progression from 7–8 cells at 18 hpf, to 5–6 at 20 hpf, leading to the characteristic configuration of compact and symmetrically organized cells, forming a unique central lumen. Additionally, we identified more cells per section and bigger section areas in proximal and distal levels compared to medial ones. The cell number per cross-section along with tubule section area was consistently higher in *han*^*s6*^ mutants at the three levels and there was also an increase in data scattering (Fig 6A and 6B).

Our measurements indicate a loss of tissue organization and compaction, evident through several abnormalities such as portions lacking cells to fully form a tubule, the formation of two smaller lumens, and aberrant localization of renal cells that do not contribute physically to the lumen at 20 and 24 hpf (Fig 6D–6F). None of these alterations were found in WT embryos (Fig 6C).

### Kidney mesonephric tissue progression monitored through histological sections

We monitored kidney mesonephric tissue progression in adult heterozygous mutants by examining histological sections to compare anatomical characteristics. We identify the general cellular composition, tubules, glomeruli, and nephrogenic clusters, allowing us to quantitatively characterize kidney regeneration.

**Fig 7:**
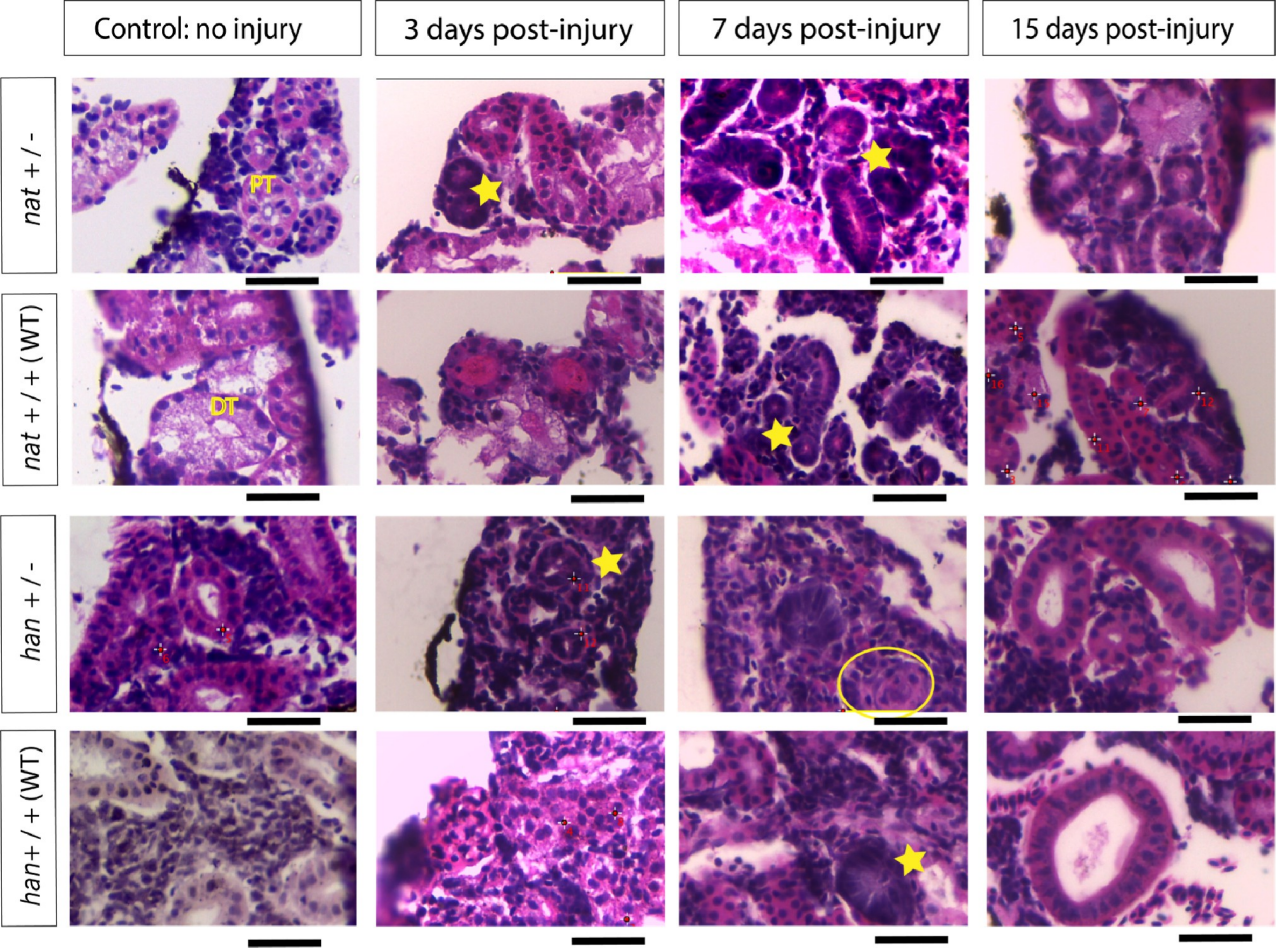
General structure of gentamicin injured zebrafish kidney tissue 3, 7 and 15 days post injury compared to uninjured control. It is possible to identify glomeruli (yellow circle); proximal tubules (PT) characterized by elongated cells, darker staining and brush border towards the lumen; distal tubules (DT) with a pink pale staining and neonephrogenic clusters that are evidence of regeneration process (yellow stars). Pink stain in lumens is an indicator of cellular debris. Scale bars (black bars) measure 0.03mm.

Section images taken up to 15 days post injury showed a similar regenerating pattern across all strains. On days 3 and 7 there was visible pink cellular debris inside lumens, altered cellular organization and densely packed nuclei in tubules, indicative of neonephrogenesis. By day 15 post-injury, tubules exhibited larger and more defined lumens, and the overall tissue structure appeared more organized, similar to control conditions. However, some sections still showed signs of neonephrogenesis, possibly indicating an ongoing regeneration process (Fig 7).

For quantitative analysis, we selected the following criteria: the number of proximal and distal tubules (identified by morphological characteristics), number of nuclei that compose these tubules, tubule lumen area, and the whole kidney section area. Initial data observation using star plots allowed us to identify differences in patterns in the measured variables compared to un-injured control. This preliminary analysis helped establish and validate these chosen criteria as appropriately changing variables during regenerative process (S5 Fig).

To further understand variable behavior and how they explained variations in kidney regeneration, we performed a Principal Component Analysis (PCA). Three main components were sufficient to explain 87% of the original variation of the data. The first two principal components revealed that the number of proximal tubules, lumen area, and nuclei in proximal tubules were the variables that best explained data variation (S6A Fig). The results also showed an inverse relationship between lumen area and proximal nuclei, as well as high covariation between total and proximal tubules, practically allowing these two factors to be combined into one variable.

Comparing the two principal components to characterize the complete tissue, we observed no significant differences between different kidney regions. That is the neck, trunk and tail of the mesonephric kidney (p>0,05) (S6B Fig).

### Adult heterozygous *nat* and *hand* mutants show an apparent mesonephric anatomical phenotype under basal (uninjured) conditions

**Fig 8:**
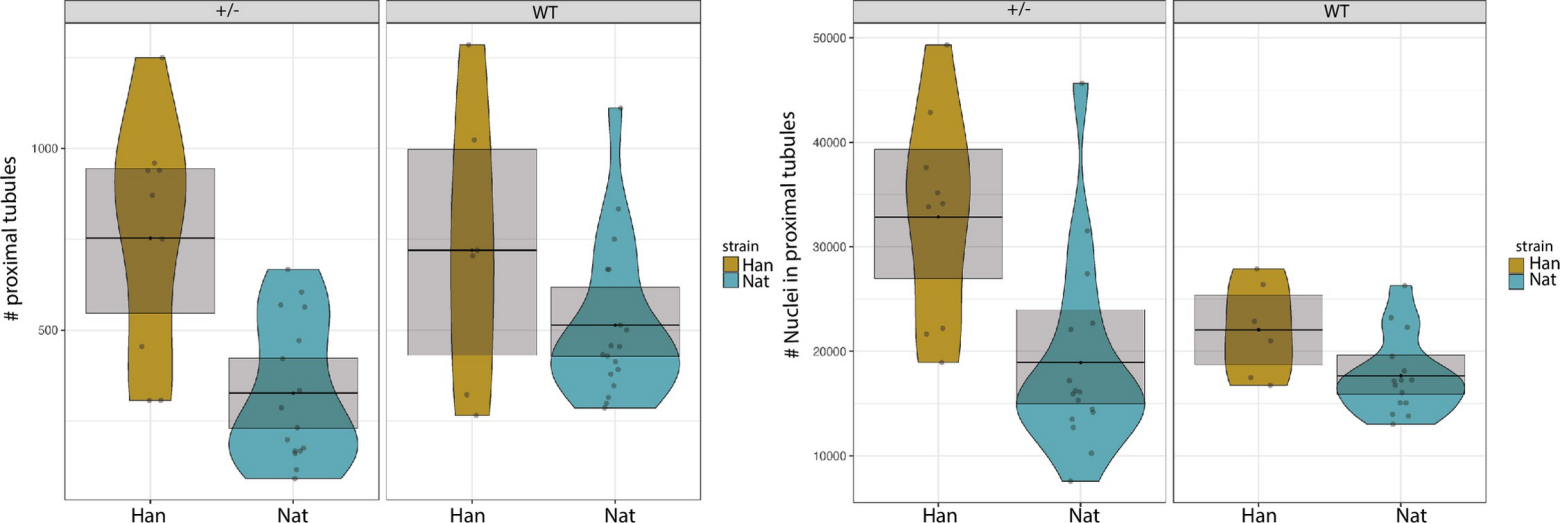
Basal tissue conditions in uninjured fish. Mixed model analysis found significant differences in number of proximal tubules (normalize by the area of tissue evaluated) and nuclei within each proximal tubule (P ≤ 0.01), with lower tubules in *nat* carriers and higher nuclei number in *han* carrier (+/-) mutants n = 3 (for each regeneration time, control and strain).

To determine the baseline cellular composition of the tissue, we compared uninjured WT, *nat*^*tl43c*^ and *han*^*s6*^ carrier mutants (Fig 8). The results showed a significantly lower number of proximal tubules in *nat*^*tl43c*^ heterozygous mutants per tissue area, and a significantly higher number of nuclei within each proximal tubule in *han*^*s6*^ heterozygous.

### Fibronectin appears to influence the anatomical composition of mesonephric tissue during regeneration

**Fig 9:**
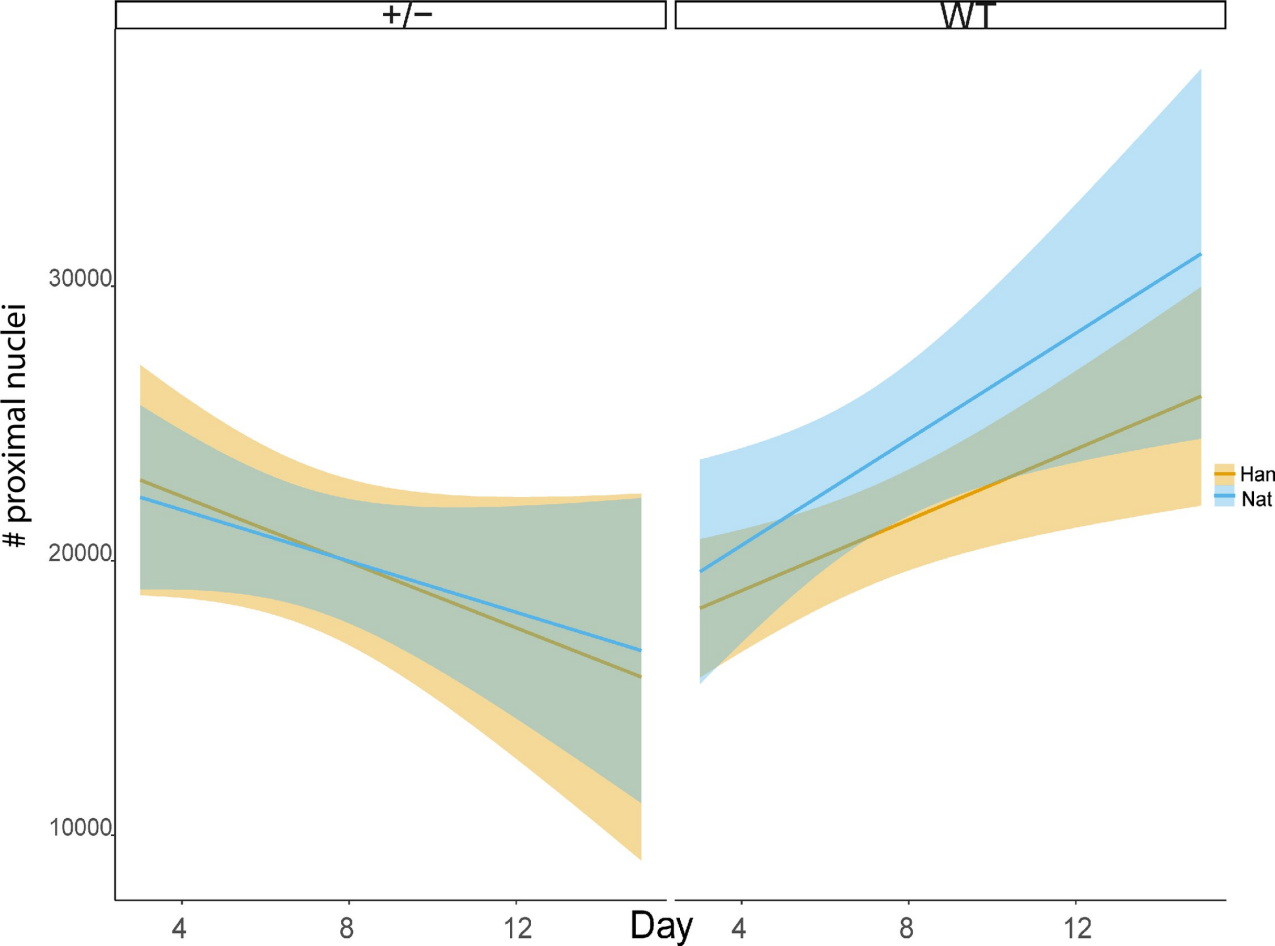
Evaluation of proximal tubule nuclei along kidney regeneration days per tubule. The lines indicate the trend, and the colored shadows represent the 95% CI confidence interval. The opposite trends show significant differences between both carriers (+/) compared to both WT (P≤0.05) n = 3 (for each regeneration time, control and strain).

Finally, we assessed if there were changes along kidney regeneration over days 3, 7 and 15 post-injury, comparing regeneration of WT tissue versus mutant tissue normalized by area of the tissue evaluated. We found difference for the variable “number of proximal tubule nuclei” between both *nat*^*tl43c*^ and *han*^*s6*^ heterozygous mutants compared to WT (Fig 9). This indicates that Fn zygosity affects specifically on the number of tubule nuclei during regeneration.

## Discussion

In this research we present novel, quantitative evidence of Fn function in renal morphogenesis and regeneration.

To start with, it is relevant to indicate that the mutant`s lack of FN is not causing any gastrulation and early mesoderm specification abnormalities likely due to maternal *fibronectin1* contribution compensating for the lack of the zygotic one at early stages. The yolk syncytial layer (YSL) functions as an important signaling center to induce the mesoderm [21]. Indeed, Mtx1 is a key YSL transcription factor that positively regulates fibronectin expression [22].

We showed that FN has a role in organogenesis of the pronephric tubules, evident in the aberrant structures seen in the histological sections of *nat*^*tl43c*^ mutants. To understand this phenomenon we established and evaluate several hypotheses based on Fn function on different organism and tissue contexts.

The role of Fn in cell state (specified, determined, differentiated) or tissue structure maintenance has been previously reported in the *nat*^*tl43c*^ zebrafish line. Somite boundaries are formed through epithelialization of mesenchymal cells from the paraxial mesoderm. This epithelialization is stabilized by the synthesis and deposition of Fn protein in the ECM, as well as by the expression of the adhesion protein N-Cadherin [23–25]. Fn is not required for the initial intersomitic boundary formation, but it is needed for their maintenance and stabilization in zebrafish [26]. Accordingly, pronephric epithelialization could be occurring while Fn protein stabilizes the architecture through signaling and mechanical support. However, in absence of this protein, specification may not be maintained over time. Our observations, did not indicate a clear effect on specification, so further evidence is needed to understand the expression of these factors during kidney morphogenesis.

We also explored the segmentation mechanism for any alteration. As shown by the expression profile of *ATP 1a*.*1*, the consistency and overlap with the reported expression of *Cdh17* gene suggests that segmentation process is occurring in both WT and mutants, independent of the lumen formation. Additional research is needed to confirm this “independent segmentation” hypothesis and understand how it could occur separately from tubulogenesis.

A possible explanation for the abnormal organization of these cells could be the lack or mislocalization of intercellular interaction protein complexes, such as tight junctions and adherent junctions, which are common in normal epithelial cells. These complexes allow cells to attach to each other and provide the structure required for normal physiology. We hypothesize that this could indicate a defect in polarization, affecting the establishment of apical and basolateral domains necessary for lumen formation. Therefore, we suggest that Fn1 plays an important role in the tubulogenesis of the pronephros and its absence could induce defects in polarization.

Furthermore, our results in *hand2* mutants confirmed previous research observations and provide evidence of FN´s role in pronephros development through a modulation mechanism, as previously reported by Garavito *et al*. in a cardiac fusion context [18].

As observed in our results, the intermediate mesoderm field size is increased, resulting in a pronephros composed of significantly more cells and wider lumens, suggesting a loss of organization and compaction within the tubule structure [20]. This effect appears to be separate from other global morphogenetic processes, given the unaffected pronephric length and otic vesicle development.

This altered phenotype of *hand2* mutants could reflect various model possibilities. As suggested by Perens *et al*. (2016) [20], *hand2* function could be cell-autonomous to limit adjacent region development or could indirectly regulate other signals that, in turn, limit these contiguous processes. Additionally, the observed phenotype could be indicative of excessive Fn deposition, as has been reported for myocardial precursors in *han*^*s6*^ embryos [11]. Overexpressed genes in the context of *hand2* loss-of-function included claudin C (*cldnc*), the Na+/k+ subunit 1a1a.4 *(atp1a1a*.*4)*, *fibronectin* (*fn1*) and its receptor integrin subunit alpha 5 (*itga5*), all linked to polarized epithelial tissue establishment or function needed in renal tubulogenesis [18]. Abnormal amounts of Fn could interfere with the required renal progenitors`rearrangement into circular clusters and the differential distribution of apical components. For instance, transmembrane proteins such as Claudins and Na+/k+ ATPase are known to have roles in generating lumens and other factors influenced by Fn might also be involved. For example, the *transcription factor 2 gene* (*tcf2)* regulates the formation of the intestinal lumen by regulating the expression of the *claudin 15* and the *Na*^*+*^*/K*^*+*^ *ATPase* genes [27]. Since the *tcf2* gene, also known as *vhnf1* (*variant hepatocyte nuclear factor 1*), is strongly expressed in the pronephros, it could also contribute to lumen formation [28]. Additionally, preliminary results from immunofluorescence of ATPase, shows that *nat*^*tl43c*^ pronephric tissue has abnormal deposition of this protein, global decrease, and cell polarity alterations.

Moreover, *hand2* loss-of-function mutants lack polarity organization, as the distinctive localization of the apico-basal markers aPKC (atypical protein kinase C) and ZO-1 (zona occludens1) is lost, failing to form myocardial epithelial monolayers [18]. Fn role in stablishing boundaries between neighboring tissues has proven important in development for cell layer patterning [26]. Lateral plate mesoderm may be contributing to boundary establishment with the intermediate mesoderm by secretion of Fn in a regulated manner. Under this hypothesis we could suggest the possibility of a signaling pathway including *hand2* as a progenitor balance effector between vascular (which shares a developmental window with nephrogenesis) and renal fate. There could likely be other upstream molecules regulating *hand2* function and activation of IM-LPM interface progenitors. These progenitors exhibit great plasticity potential, evidenced by parallel pathways that could also influence this process and that act as independent networks, such as the zinc-fingers transcription factor: *osr1*, which exerts a functional antagonism with *hand2*, also influencing renal and vascular differentiation. Although the mechanism by which the transcription factor regulates ECM components has not been described Firulli`s lab has explored molecular mechanisms of bHLH transcription factors specifically contributing to heart development. It has been reported that Hand2 function can be affected by dimerization affinity, adjustments of gene expression levels, or posttranscriptional regulation trough microRNAs. His work on these transcription factors’ regulation could guide research on different tissues such as the kidney [29, 30].

Having made these observations that contribute to unraveling the importance of Fn as an extracellular matrix component in pronephros morphogenesis, we wanted to observe its possible influence in nephroneogenesis in mesonephros tissue regeneration. The extracellular matrix is particularly important because it creates an adequate environment that can stimulate cellular reconstruction and establishment of new functional tissue.

It has been established mesonephros has populations of tissue-resident progenitor cells that form nephrogenic aggregates and differentiate into nephrons following a developmental-like process. This implies that zebrafish kidney regeneration occurs while some tubular epithelium and renal structures remain [31–33]. We were able to see visual evidence of this process by identifying higher nuclei number and undefined smaller lumens during earliest timepoints after injury, showing also that gentamicin is not causing a massive damage, leaving residual material for regeneration to occur.

Before analyzing regenerative changes, we characterized basal (uninjured) tissue conditions, revealing unprecedented evidence of different phenotypes in heterozygous *nat*^*tl43c*^ and *han*^*s6*^ mutants in homeostatic conditions. For two of our measured variables there were significant differences, revealing a smaller number of proximal tubules in heterozygous *nat*^*tl43c*^ mutants and higher tubule nuclei number of *han*^*s6*^ heterozygous in the control, uninjured tissue.

These phenotypes match our evidence obtained for homozygous mutants in embryogenesis such as higher cellularity reported also in *han* mutant embryo´s tubules. These basal condition results show once again the influence of Hand2 as an important regulator of kidney development and regeneration. Further experiments are needed to corroborate the origin of these mutant phenotypes which could be a consequence of direct and indirect functions altering a more complex signaling pathway, probably involving other molecules such as ECM components or other regulation identity or differentiation processes.

Trinh & Stainier (2004) [11], previously determined that heterozygous embryos had a WT phenotype. However, our results may indicate a possible haploinsufficiency scenario, where some variables, possibly through genetic compensation, are unaffected, while others are affected by the presence of only one copy of the gene. This phenomenon can be related to haploinsufficiency “dosage-stabilizing” hypothesis. According to this, haploinsufficiency is a balance mechanism for a gene product (usually an important protein like Fn), where its dosage is limiting for several biological processes, and detrimental when overproduced [34]. This scenario aligns with the results observed in both *nat*^*tl43c*^ (*fn* underexpression) and *han*^*s6*^ (*fn* overexpression) mutants.

Another main finding reveals significant differences between carrier mutants and WT individuals during regeneration, suggesting that their heterozygous condition could affect one or several processes during tissue repair. Among our chosen variables, the number of proximal tubule nuclei was the main factor distinguishing regeneration in heterozygous individuals compared to WT. These differences could indicate Fn’s role in kidney repair, suggesting that regeneration might follow the paradigm of recapitulating developmental processes. It is possible that Fn undergoes dynamic regulation and deposition after inducing kidney injury, influencing regenerative events and contributing to the consolidation of epithelial structure.

As previously mentioned, Fn could have various functions and might play tissue-specific roles. This role depends on the morphogenetic conditions of each organ. For instance, kidney regeneration in zebrafish recapitulates mesonephros development, in which progenitor cells accumulate and associate to distal tubules, forming aggregates that elongate in a tubular epithelium, differentiating to form a functional nephron [3, 6, 32]. To date, some factors have been reported as critical for kidney regeneration in zebrafish. One of these is fibroblast growth factor (FGF), which is induced after renal injury and is necessary to recruit progenitor cells to nephron formation sites [32]. FGF´s distribution is matrix-dependent, and Fn has been indicated to influence the diffusion of this growth factor [18]. This could be one of the mechanisms through which Fn influences tissue composition.

Understanding basic tissue arrangement and specific regeneration conditions helps identify relevant regulatory signals or factors for each regenerative context. Although heterozygous mutants do not exhibit a full loss of function phenotype, it was possible to observe that after an injury, heterozygous fish likely cannot fully activate developmental and regenerative-specific pathways.

This study presents a novel approach and variables to assess zebrafish kidney development and regeneration identifying Fn´s role. This contributes to identifying of important factors that can promote regeneration in humans or better understand tissue morphogenesis dynamics.

Additionally, we present new evidence validating the zebrafish model as an emerging acute kidney injury model and revealing the importance of ECM in another tissue-regenerating context.

Overall, our experimental approach offers novel insights and perspectives on the effects of *hand2* in renal organogenesis at the cellular level, emphasizing its relevance in preserving progenitors’ boundaries and regulating morphogenetic features such as tissue organization and structure, which may depend on polarization.

## Methods

### Zebrafish strains and husbandry

Adult zebrafish were raised at 28,5°C on a 14 hours light/ 10 hours dark cycles under controlled water parameters: conductivity 600–800 uS/cm, pH 7,0–7,6 and oxygen supplementation 6–8 ppm. All living fish handling, breeding and embryo collection were done in compliance with official Universidad de los Andes IACUC standard operative procedures -SOPs-: 15_004; 15_005; 15_006; 15_007. All procedures were revised and approved by the Institutional Animal Care and Use Committee (IACUCs) from the Universidad de Los Andes through the Animal use Format (FUA) C.FUA_17–004.

Lines *han*^*S6*^ and *nat*^*t*l43c^ obey to autosomal recessive pattern mutation in *Hand2* and *fibronectin 1* gene respectively. *Han*^*s6*^ line was provided by Dr. Deborah Yelon (University of California, San Diego). *han*^*S6*^ mutant embryos were obtained from crossings of heterozygous *han*^*S6*^ fish [19]. Embryo clutches were desensitized by exposure to chilled PBS, and fixed at 18, 20 and 24 hpf (*han*^*S6*^ embryos) and 18, 21, 24, 32 and 36hpf (*nat*^*tl43c*^) according to previously reported procedures [35]. Fixation was made using 4% PFA/PBS overnight (ON). Embryos for whole mount *in situ* hybridization were rinsed in PBS and dehydrated in methanol and storage at -20ºC until use.

### Whole mount in situ hybridization

Chromogenic one and two-color whole-mount in situ hybridizations were performed as previously described [36, 37]. We used the following probes synthesized from linearized plasmid templates: *atp1a1a*.*4* (ZDB-GENE-001212-4), *myl7* (ZDB-GENE-991019-3), *kdrl* (ZFIN ID: ZDB-GENE-000705-1), *pax2a* [38], *myl7* [19] and *ATPα1a*.*1* (ZIRC #:708).

After detecting the expression patterns of interest, embryos were rinsed in PBS-Tween 0,1%, refixed with PFA 4% ON, washed and dehydrated in Methanol. To carry out macroscopic observations and measurements, stained embryos were cleared in 2:1 Benzyl benzoate: Benzyl alcohol solution and mounted on depression slides.

### Histological processing

*Hand2* mutant and WT embryo`s histological analyses were performed on WISH-processed embryos embedded in Spurr-Low Viscosity infiltrating mixture (SPI) as previously reported [20], with the following modifications: Embryos were rehydrated after Methanol incubation post *WISH* staining through a PBS buffer gradient. Samples were oriented as desired and incubated for 18–20 hours at 60°C for resin polymerization.

4 um transverse sections were cut with glass blades, collected in glass slides and stained with basic fuchsine (1 mg/ml in 5% ethanol) for 20–30 seconds at 65°C, similar to reported staining protocols [39].

*Nat*^*tl43c*^ embryos and adult`s renal tissues were fixed in 4% paraformaldehyde for at least five hours at room temperature. Later they were dehydrated in an increasing ramp of ethanol followed by Isopropanol and embedding in paraffin by increasing paraffin-xylene solutions. Cross sections 4 μm thick were made with a Leica microtome along the anterior-posterior axis. Sections were deparaffinized by heat and xylene rinse. Sections were then rehydrated and stained with hematoxylin/eosin. Same procedure was applied to hybridized embryos after post-fixing in paraformaldehyde.

### Gentamicin injections

Zebrafish were injured through a standardized lesion process using gentamicin (nephrotoxic antibiotic) intraperitoneal injections. Fish were first anesthetized using 0,016% Tricaine and weighted. Gentamicin was administered at a concentration of 100mg/kg in peritoneal cavity. Injured individuals were verified by detecting white casts of dead epithelial kidney tissue in the water, excreted overnight or two days post-injection [40]. Detailed protocol is included in S7 Fig.

### Zebrafish adult kidney dissection

Zebrafish adult kidney dissection was done on euthanized individuals by overdose of 0,2% Tricaine concentration. Afterwards, the head was removed and an incision in peritoneal cavity was made to be able to remove organs and expose the kidney, cutting off excess skin to facilitate manipulation [41]. Fixation was made using a 4% paraformaldehyde (PFA) / PBST1x and 0,1% of dimethyl sulfoxide (DMSO) solution. Euthanized animals were submerged in the solution overnight at 4ºC. Finally, PFA is removed and replaced with PBST 1x to extract the organ with fine forceps [41].

Kidney samples were collected in days 3, 7 and 15 post-injection for further histological and *in situ* hybridization analysis. Sample number is explained in detail in S1 Table.

During kidney dissection, the “head” region could not be removed or was destroyed, for this reason, we included a new kidney anatomical region we called “neck” thereafter.

### Imaging

#### Whole mount embryos

Images of cleared whole embryos were captured with a Leica MC170 HD camera on a Leica MC165 FC stereoscope.

#### Transverse sections of epoxy embedded embryos

RGB images were acquired with a *Zeiss* Axioskop 2 Microscope coupled to an *Infinity* camera set. Optimization of image quality was performed applying RGB color decomposition and image calculator tools on FIJI software package.

Measurements and quantitative analysis in general, were performed using FIJI and ImageJ software package.

### Statistical analysis

Normal distribution and data variance homogeneity of measured morphometric parameters were tested using JASP software package. In case of violations of these assumptions, data transformation or non-parametric equivalent test for ANOVA were conducted. When found statistical significance among tested categories, *post hoc* -Tukey or Dunn- tests were applied to determine differences between pairs of categories (*wild-type* vs. *han*^*s6*^ individuals). All indicated n number for each experiment corresponds to total embryos tested per condition (S2 Table).

For the adult tissue section analysis, several representative sections of the same organ (same individual) were processed. For this reason, we performed mixed models. To evaluate regeneration and possible differences between cellular conditions in mutant carrier´s kidneys, they were compared to WT, a principal component analysis was performed to reduce redundancy in the measured variables. These statistical analyses were made using software R.

## Supporting information

S1 Fig Cell count in identified pronephric structures. The number of cells composing a pronephric structure was determined as examined in transversal slides H&E histology and at three distinctive anatomical locations (proximal, medial, distal). Pronephric cell counts are lower in mutants at all the levels examined. There is an increased tendency but not significant at the distal level and over time. n = 8. Count was done on two independent histological series. Results presented as mean ± s.e.m. p<0.05.
(PDF)

S2 Fig Pronephric tubule length comparison in *nat^tl43c^* mutants. Only by 48 hpf there is a significant increase in mutants. n = 8.
(PDF)

S3 Fig Pronephric tubule length comparison at 20 and 24 hpf in *han^S6^* mutants. No significant differences were detected at evaluated stages (n = 7, p> = 0.005; Dunn’s post hoc test).
(PDF)

S4 Fig Morphoanatomical differences. Differences in vascular vessel and pronephric morphogenesis between WT and *han^S6^* at 18 hpf.(left) The global location of endothelial fated cells is equivalent between WT and *han^S6^*. A scattered pattern of expression of *kdrl* is observed in *han^S6^* embryos, suggesting angioblasts mis localization or, ectopic-aberrant expression by non-angioblast fated cells (right). Black hollow arrows indicate the approximate location of tissue section in the A-P axis. Dilation of pronephric tubule is confirmed, along with augmented tubule cell number. Scale bars: whole embryos: 200 μm; sections: 20 μm.
(PDF)

S5 Fig Starplots showing standardized data. Data for all the measured variables in *nat^tl43c^* and *han^S6^* heterozygous compared to WT (+/+). Bigger circumference shows higher mean value in each variable.
(PDF)

S6 Fig A) PCA analysis showing two main components. PC1 is mainly explained by proximal tubule number and PC2 is mainly explained by Lumen Area and Proximal Nuclei which have an inverse relationship. B) Comparison between kidney regions (neck, tail and trunk). PC1 is mainly explained by number of proximal tubules while PC2 summarizes lumen area and nuclei in proximal tubules. Confidence intervals (95%) represented by the gray boxes overlapping show no significant differences.
(PDF)

S7 Fig Gentamicin injection protocol. (PDF)

S1 Table Sample number for adult kidney tissue experiments. (PDF)

S2 Table Spread sheet containing quantitative raw data. (XLSX)
