# Correction: Fibronectin and Hand2 influence tubulogenesis during pronephros development and mesonephros regeneration in zebrafish (Danio rerio)

**DOI:** 10.1371/journal.pone.0328807

**Published:** 2025-07-21

**Authors:** Lucia Carolina Uribe-Montes, Camilo Alfonso Sanabria-Camargo, Cristian Camilo Piñeros-Romero, Sebastián Otálora-Tarazona, Estefanía Ávila-Jiménez, Edwin Acosta-Virgüez, Zayra Viviana Garavito-Aguilar

The following information is missing from the Funding statement: ZV. Garavito-Aguilar (Universidad de los Andes – The Office of the Vice-president of Research and Knowledge Creation – Publication and Exhibition Grant CPE-0719-24).

The full Funding Statement is as follows: L. Uribe-Montes (Universidad de los Andes – Science School Grant for Master’s Students INV-2018-34-1286) C. Sanabria-Camargo (Universidad de los Andes – Science School Grant for Master’s Students:INV-2019-85-1836) C. Piñeros-Romero (Universidad de los Andes – Science School Grant for Master’s Students INV-2017–2) Estefania Ávila-Jiménez (Young Researcher for grant MinCiencias for basic sciences [852 CD 1204-852-70312) ZV. Garavito-Aguilar (Science School Program to Faculties Grant INV-2021-128-2322) ZV. Garavito-Aguilar and E. Acosta-Virgüez (grant MinCiencias for basic sciences [852 CD 1204-852-70312]. ZV. Garavito-Aguilar (Universidad de los Andes – The Office of the Vice-president of Research and Knowledge Creation – Publication and Exhibition Grant CPE-0719-24).
