# Supplementary figures and images for: Fibronectin and Hand2 influence tubulogenesis during pronephros development and mesonephros regeneration in zebrafish (Danio rerio)
Source: PLoS One. 2024 Sep 6;19(9):e0307390. doi: 10.1371/journal.pone.0307390 (PMC11379296; doi:10.1371/journal.pone.0307390)

**A**

**Number of pronephric cells**  
**Proximal Region**

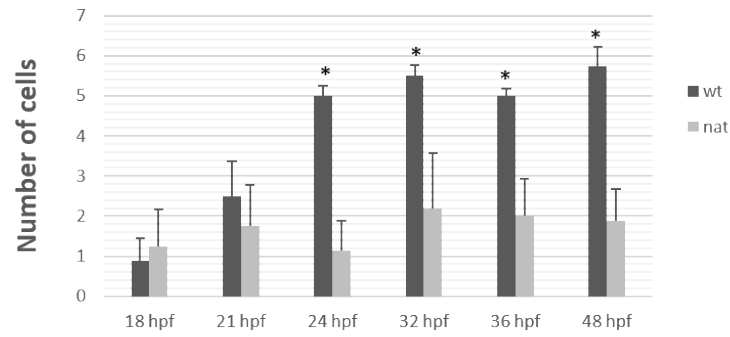

**B**

**Medial Region**

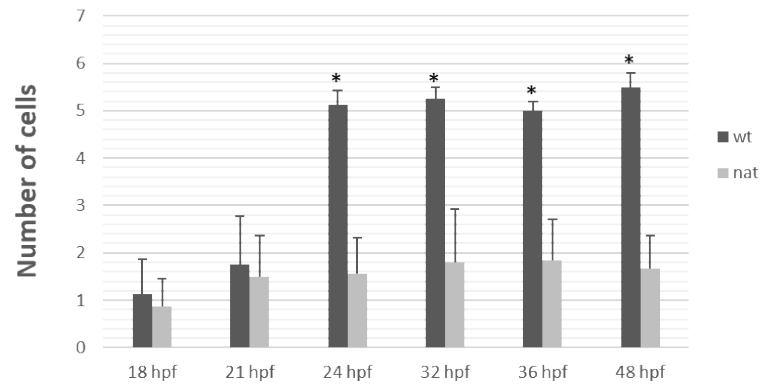

**C**

**Distal Region**

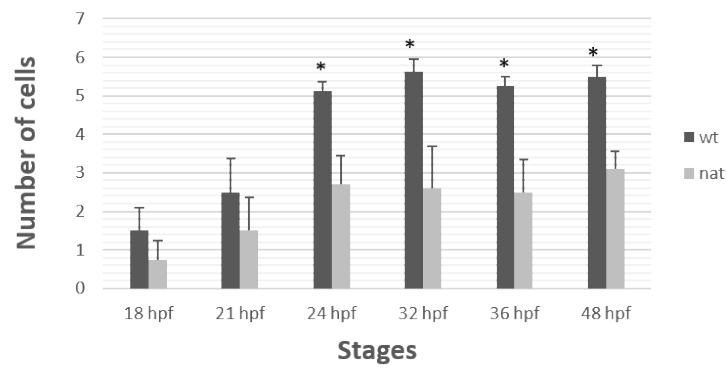

Supplement: S1 Fig — The number of cells composing a pronephric structure was determined as examined in transversal slides H&E histology and at three distinctive anatomical locations (proximal, medial, distal). Pronephric cell counts are lower in mutants at all the levels examined. There is an increased tendency but not significant at the distal level and over time. n = 8. Count was done on two independent histological series. Results presented as mean ± s.e.m. p<0.05. (PDF) [file pone.0307390.s001.pdf]

### Pronephric cord antero-posterior length

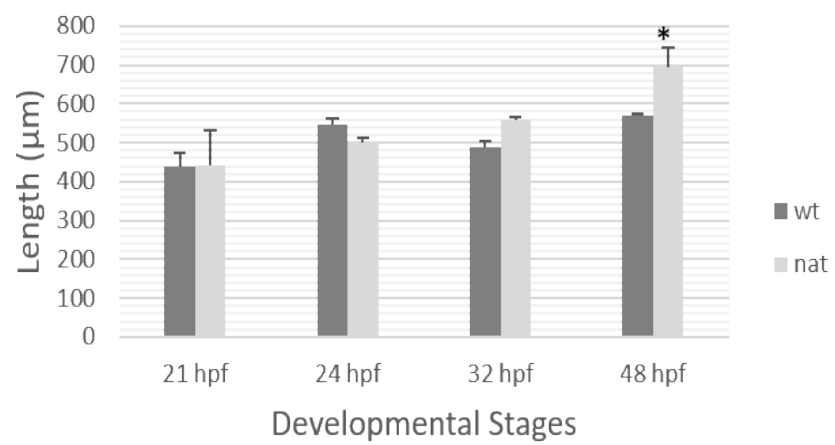

Supplement: S2 Fig — Only by 48 hpf there is a significant increase in mutants. n = 8. (PDF) [file pone.0307390.s002.pdf]

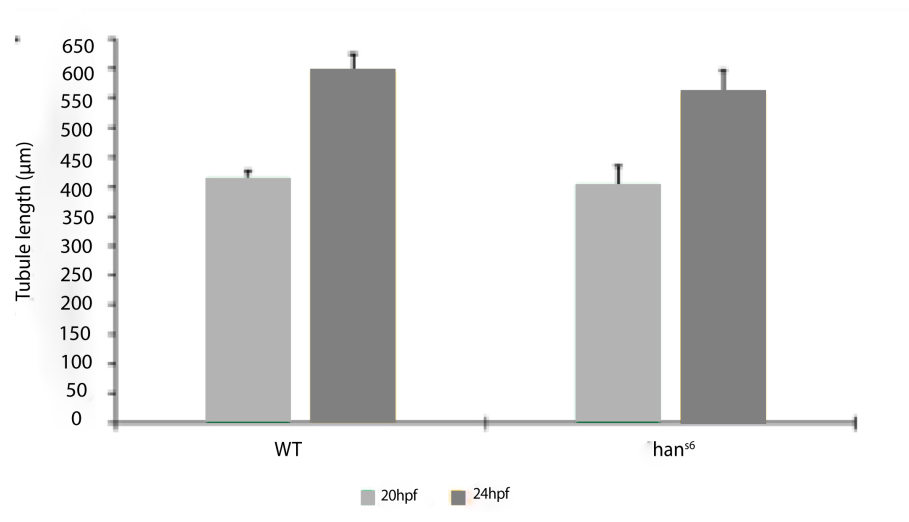

Supplement: S3 Fig — No significant differences were detected at evaluated stages (n = 7, p> = 0.005; Dunn’s post hoc test). (PDF) [file pone.0307390.s003.pdf]

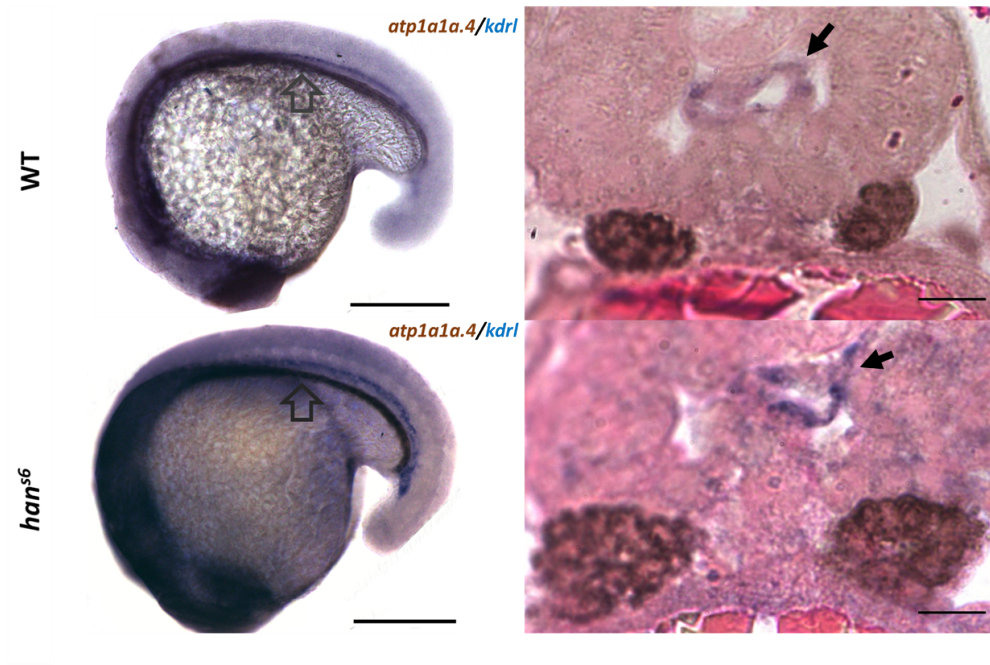

Supplement: S4 Fig — Differences in vascular vessel and pronephric morphogenesis between WT and hanS6 at 18 hpf.(left) The global location of endothelial fated cells is equivalent between WT and hanS6. A scattered pattern of expression of kdrl is observed in hanS6 embryos, suggesting angioblasts mis localization or, ectopic-aberrant expression by non-angioblast fated cells (right). Black hollow arrows indicate the approximate location of tissue section in the A-P axis. Dilation of pronephric tubule is confirmed, along with augmented tubule cell number. Scale bars: whole embryos: 200 μm; sections: 20 μm. (PDF) [file pone.0307390.s004.pdf]

Values

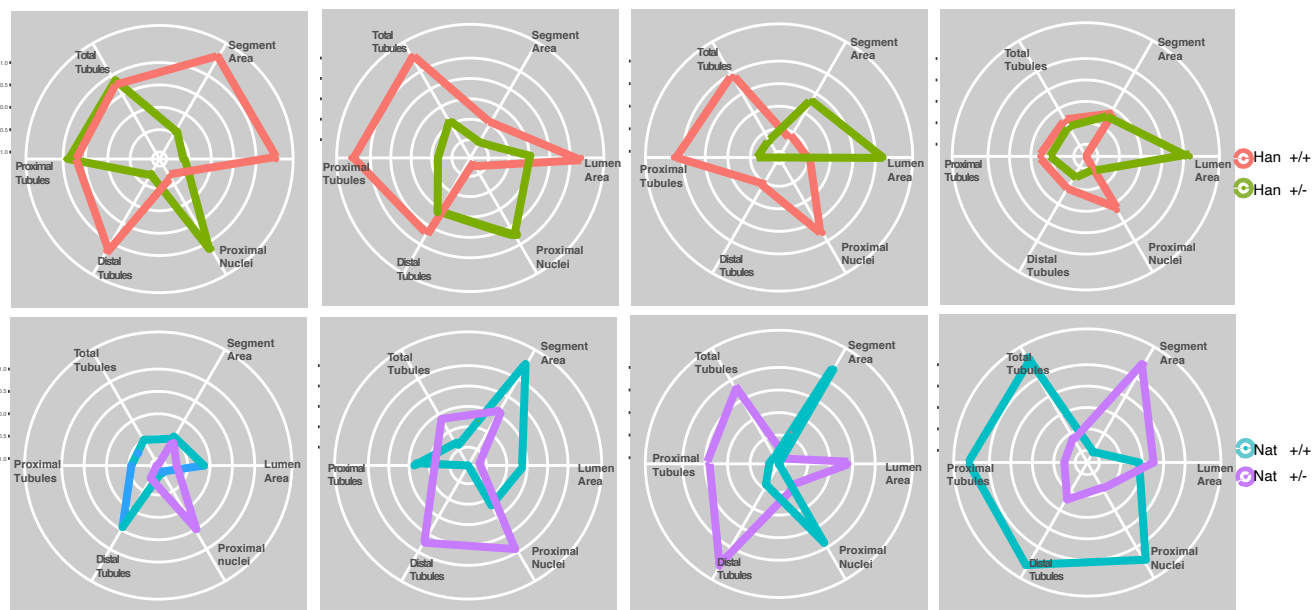

Criteria

Supplement: S5 Fig — Data for all the measured variables in nattl43c and hanS6 heterozygous compared to WT (+/+). Bigger circumference shows higher mean value in each variable. (PDF) [file pone.0307390.s005.pdf]

A

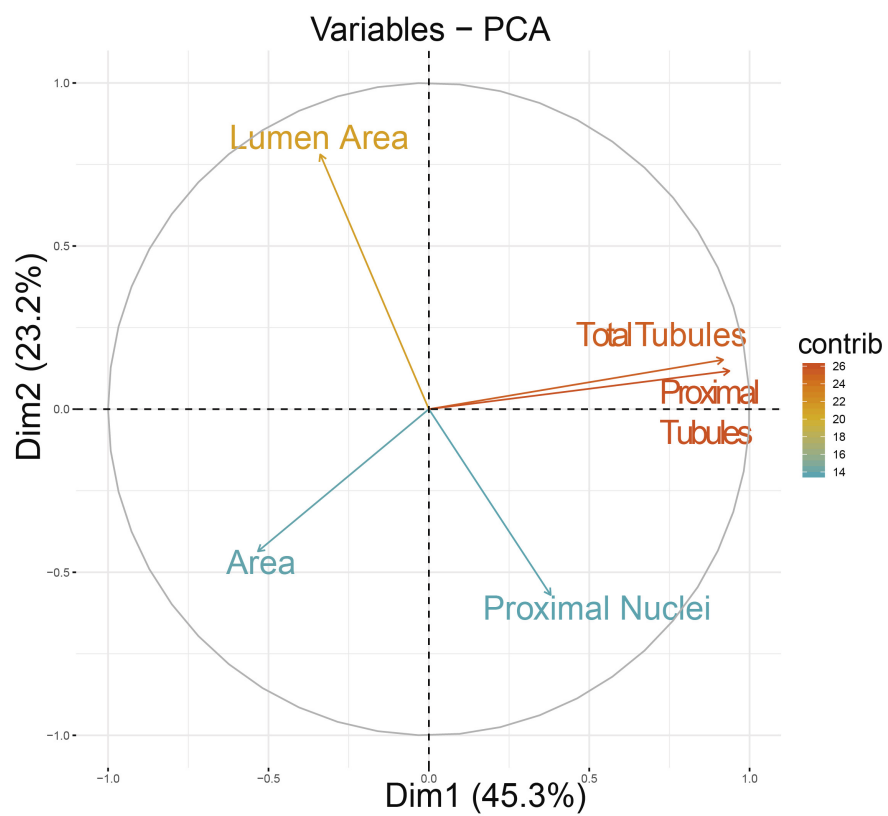

B

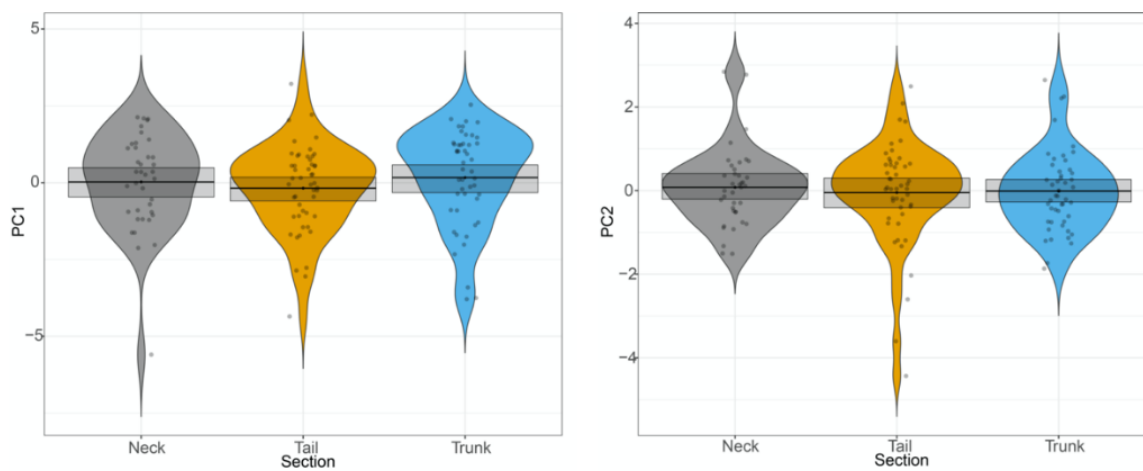

Supplement: S6 Fig — A) PCA analysis showing two main components. PC1 is mainly explained by proximal tubule number and PC2 is mainly explained by Lumen Area and Proximal Nuclei which have an inverse relationship. B) Comparison between kidney regions (neck, tail and trunk). PC1 is mainly explained by number of proximal tubules while PC2 summarizes lumen area and nuclei in proximal tubules. Confidence intervals (95%) represented by the gray boxes overlapping show no significant differences. (PDF) [file pone.0307390.s006.pdf]
