# Supplementary material for: Fibronectin and Hand2 influence tubulogenesis during pronephros development and mesonephros regeneration in zebrafish (Danio rerio)
Source: PLoS One. 2024 Sep 6;19(9):e0307390. doi: 10.1371/journal.pone.0307390 (PMC11379296; doi:10.1371/journal.pone.0307390)
Supplement: S7 Fig — (PDF) [file pone.0307390.s007.pdf]

Gentamicin injection

Materials:

- Sigma gentamicin sulfate salt 50MG (CAS Number 1405-41-0)
- Insulin syringe
- Physiologycal serum

Prepare stock solution using miliQ water. Prepare further dilutions with physiological serum.
Concentration: 100mg/Kg (fish weight)
Maximum volume for injection: 20µl

- Anesthetize fish using tricaine 0,016%, weight fish using scale with a wet towel.
- Take fish into your hands and inject adequate volume of gentamicin into intraperitoneal space with a 45º angle to avoid causing damage to internal organs.
- Immediately place injected fish into a separate tank to monitor its reaction and effectiveness of injury (white casts in the tank can appear up to the second day post injection).
