# Supplementary material for: Fibronectin and Hand2 influence tubulogenesis during pronephros development and mesonephros regeneration in zebrafish (Danio rerio)
Source: PLoS One. 2024 Sep 6;19(9):e0307390. doi: 10.1371/journal.pone.0307390 (PMC11379296; doi:10.1371/journal.pone.0307390)
Supplement: S1 Table — (PDF) [file pone.0307390.s008.pdf]

Supplementary Figure 7. Sample number for adult kidney tissue experiments

**Strain**	**Regeneration Day**	**Number of Individuals (sample size)**	**Number of sections analyzed** 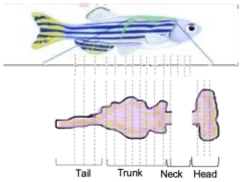
Nat	Control (no injury)	3	16
	3	3	9
	7	3	16
	15	3	9
Han	Control (no injury)	3	9
	3	3	9
	7	3	9
	15	3	9
WT	Control (no injury)	4	21
	3	4	12
	7	3	12
	15	3	9
